# Latent profile analysis on the effectiveness of tutor performance: Influence on medical students’ engagement in blended problem-based learning

**DOI:** 10.1371/journal.pone.0292843

**Published:** 2023-10-13

**Authors:** Heoncheol Yun, Seon Kim, Eui-Ryoung Han

**Affiliations:** 1 Institutional Research Center, Chosun University, Gwangju, South Korea; 2 Department of Medical Education, Chonnam National University Medical School, Gwangju, South Korea; Satyawati College (Eve.), University of Delhi, INDIA

## Abstract

Tutor performance is a critical factor for the success of a problem-based learning (PBL) curriculum. This study investigated medical students’ perceptions of the effectiveness of tutor performance and the relationships with learning engagement (behavioral, emotional, and cognitive engagement) in the blended PBL approach. This study employed a cross-sectional survey and data were collected from 238 first-year and second-year medical students. Latent profile analysis (LPA) was used to investigate how individual students’ perceptions of tutor performance can be clustered. Follow-up multiple one-way analyses of covariance (ANCOVA) were performed to examine the relationships with students’ learning engagement in PBL activities. The effectiveness of tutor performance perceived by students was classified into lowly, moderately, and highly effective tutors. These clustering profiles were significantly related to the different types of learning engagement in the blended PBL process. Hence, this study highlights that the tutor is one of the key aspects of assessing the implementation of blended PBL since student performance is positively associated with the perception of tutor effectiveness.

## Introduction

Problem-based learning (PBL) is a pedagogical methodology commonly applied for collaborative and active learning in a medical education context. Key features of the successful PBL process are the use of authentic learning groups and tutors as facilitators [1]. In PBL, a tutor in the group sessions enables students to identify problematic situations, brainstorm key concepts and ideas, research and share potential solutions, and reflect on their learning processes [2]. As the demand for distance education is gradually increasing in response to the needs for flexible learning environments, including online learning during COVID-19 pandemic, a blended PBL approach has been implemented as a complementary pedagogical tool, while enhancing the intrinsic PBL attributes discussed in previous research [3–5]. Blended learning refers to a combination of face-to-face and online learning instructions, resulting in an integrated pedagogical approach that supports each other in maximizing student learning outcomes [6,7]. Although pure online learning provides limited social contact, the blended learning approach stems from the idea that learning is essentially a social process [8]. Therefore, the blended PBL approach supports the constructivist theory of learning, where learning occurs in a social context through collaborations, negotiations, debate, and peer review [5].

While distance education provides more flexible learning environments and the students have more autonomy in making decisions regarding their learning, they might be isolated from the learning community [9,10]. However, the potential benefits of blended learning enhance pedagogy, which represents a combination of learning theories such as student-centered approaches based on constructivism and instructor-led approaches based on behaviorist principles [6]. Substantially, blended PBL increases students’ engagement and satisfaction and allows for more flexibility in organizing the PBL process [7,11]. In this sense, to affect learning in blended PBL activities, tutors as facilitators must understand and implement their supportive roles [12]. Specifically, PBL tutors need to address what the groups require in the PBL process, manage group dynamics by highlighting social congruence, and facilitate group performance via engagement strategies [13–15]. Furthermore, Donnelly [16] reported that tutor performance in the blended PBL environment contributed to motivating academic practice and promoting collaborative work with authentic PBL modules. On the other hand, there have been several challenges such as misunderstanding the educational objectives of PBL, inadequate facilitation skills, and insufficient tutor guides that may hinder tutors’ performance and effectiveness [17]. Hence, medical schools should provide continuous needs-based professional development to ensure the quality of prospective PBL tutors’ performance through reliable tutor assessment [4,18].

Ideally, to support student learning, blended PBL tutors should encourage students to engage in the PBL process through effective tutoring. In academic settings, learning engagement refers to the quality of effort students make to accomplish desired learning objectives [19]. Empirical evidence indicates that engagement in medical education plays a significant role in the learning performance of medical students and instructional effectiveness [20,21]. Furthermore, self-efficacy is another factor that affects student motivation and self-regulated learning skills during PBL [22]. According to the literature, the importance of tutors’ roles and performance was rigorously emphasized in supporting student engagement and self-efficacy in PBL.

Numerous studies enumerate the effects of PBL methodology on learning outcomes using a variable-centered approach, such as knowledge retention [23], collaboration [24], student satisfaction [25], and problem-solving and self-regulated learning [26]. That is, many studies have highlighted the relationships between student performance in PBL as predictor variables and learning achievement as outcome variables [18]. However, this approach may be weak in identifying common attributes of individuals across heterogenous groups that affect outcome variables [27]. Thus, latent profile analysis (LPA) as a person-centered approach is more adequate to explore the complex structure of tutor performance effectiveness in the blended PBL. In fact, prior research rarely focused on individual medical students’ perceptions or evaluated the effectiveness of tutor performance using a person-centered approach, which are associated with learning outcomes and support tutor development [12,18,28].

Consequently, this study assumed that medical students may not equally perceive the effectiveness of tutor performance in stimulating constructive, self-directed, contextual, collaborative learning, and the tutor’s interpersonal behavior. Still, little research has been conducted to examine the extent to which individual medical students perceive the effectiveness of tutor performance associated with learning engagement in the blended PBL. We aimed to investigate the classification of tutor effectiveness based on medical students’ perceptions of their performance profiles and the relationship with learning engagement in the blended PBL. We addressed the following research questions: 1) how can the effectiveness of tutors in the blended PBL be classified based on medical students’ perceptions of their performance? and 2) how is the effectiveness of tutors in the blended PBL related to learning engagement after controlling for performance self-efficacy?

## Methods

### Research design, participants, and setting

The current study used a cross-sectional survey which is the suitable way to study the associations of the effectiveness profiles of tutor performance with learning outcomes [29]. This study was conducted at the end of the second semester in December 2021 at Chonnam National University Medical School (CNUMS) in South Korea. After obtaining informed consent for participation in this study, 241 medical students were asked to voluntarily complete a questionnaire. After removing three cases due to incomplete survey responses, the data collected from 238 students were used for further analyses. These participants consisted of 114 (47.9%) first-year and 124 (52.1%) second-year medical students. They included 84 (35.3%) female and 154 (64.7%) male students with a mean age of 22.53 (SD = 1.34).

The PBL program at CNUMS consisted of two two-hour sessions per PBL tutorial and a one-hour colloquium. All medical students were divided into 14 small groups and participated in a total of six PBL modules. Each tutorial group had a maximum of nine students and one tutor. PBL tutors were obligated to attend a faculty development seminar focusing on the principles of PBL and tutoring, such as creating a motivating environment for self-directed learning, clarifying learning objectives, asking open-ended questions to stimulate critical thinking, encouraging group dynamics, and providing constructive feedback [30]. During the first PBL tutorial, tutors facilitated small group discussions to identify problems, generate hypotheses, formulate plans to resolve problems, and identify learning issues to achieve learning goals. Between the tutorials, students searched for resources and information to understand important concepts. In the second tutorial the following week, PBL tutors encouraged students to ask each other questions, explain difficult concepts to each other, and apply new concepts to the PBL module.

CNUMS adopted blended PBL utilizing the combined features of both instructional modalities through the learning management system (LMS), such as synchronous and asynchronous approaches [31]. The first-year PBL course was held from the third week of August to the last week of November, and the second year was from the first week of October to the last week of November. Due to social distancing policies during the COVID-19 pandemic in August through October, the first four PBL modules in the first year included synchronous sessions administered via web-based videoconferencing platforms such as ZOOM and asynchronous sessions conducted via discussion forums in the LMS, but the last two PBL modules were conducted in-person. The first two PBL modules of the second year were online classes and the last four were in-person classes. Clinical presentation of the first year PBL modules included obesity, fever, weight gain, chest discomfort, cough, and dyspnea and those of the second year were hypotension, cough, abdominal pain, headache, fever, and weight loss.

### Measures

To measure the effectiveness of blended PBL tutor performance, we used the short tutor evaluation instrument, developed and validated by Dolmans and Ginns [32]. Originally, this instrument was developed for students to collect valid and reliable information about the performance of tutors in PBL settings [32]. Students should be asked to complete this short questionnaire on a regular basis that is less burdensome to them [32]. We modified the Korean version of the short tutor evaluation instrument used [33]. This tutor evaluation instrument includes 11 items with five underlying factors: constructive learning, self-directed learning, contextual learning, collaborative learning, and the tutor’s interpersonal behavior [34]. All tutor performance evaluation items were rated using a 5-point Likert scale ranging from 1 = *strongly disagree* to 5 = *strongly agree*. Reliability coefficients using Cronbach’s alpha were sufficiently high, as shown in Table 1.

**Table 1:** Subscales and reliability coefficients of tutor effectiveness and learning engagement.

Subscale (no. of items)	Description (sample item)	Cronbach’s alpha
*Tutor Effectiveness* (11)		
Constructive learning (3)	Tutors stimulate students to construct their own knowledge (The tutor stimulated us to summarize what we had learnt in our own words.).	.914
Self-directed learning (2)	Tutors stimulate students to plan, monitor, evaluate, and reflect on the learning process (The tutor stimulated us to generate clear learning issues by ourselves.).	.846
Contextual learning (2)	Tutors stimulate students to be exposed to relevant cases or problems (The tutor stimulated us to apply knowledge to the problem discussed.).	.838
Collaborative learning (2)	Tutors stimulate students to develop and share alternative perspectives, and interact with them. (The tutor stimulated us to give constructive feedback about our group work.)	.820
Interpersonal behavior (2)	Tutors stimulate students to be involved in an adequate effective climate among them (The tutor was clearly motivated to fulfil the role of facilitating small group learning.).	.796
*Learning Engagement* (19)		
Behavioral engagement (5)	Behavioral engagement entails positive learning conduct and participation in school activities. (I complete my homework on time.)	.706
Emotional engagement (6)	Emotional engagement refers to student’s affective reactions to learning (I am interested in the work at the blended PBL class.).	.809
Cognitive engagement (8)	Cognitive engagement includes psychological investment and flexibility in learning (When I read the course materials, I ask myself questions to make sure I understand what it is about.).	.873

Medical students’ learning engagement was measured with 19 items of engagement, adopted from Sun and Rueda [19]. Specifically, learning engagement was categorized as behavioral, emotional, and cognitive engagement associated with motivation and learning performance [35,36]. After the instrument validation, Sun and Rueda [19] investigated the relationship between motivational and learning factors in distance education settings. To assess medical students’ learning engagement, we modified the Korean version of learning engagement [37]. Participants responded to the engagement items with a 5-point Likert scale (1 = *strongly disagree* to 5 = *strongly agree*) and reliability coefficients using Cronbach’s alpha were moderately acceptable, as presented in Table 1.

Self-efficacy refers to a student’s judgment of their capability to implement a specific learning activity while dealing with a difficult task [38]. To assess medical students’ self-efficacy, we used the item “how confident are you that you will be able to handle the difficulties in the upcoming exam?” in the affective learning survey instrument [39], rating judgment on a scale ranging from 1 = *not at all* to 9 = *very*.

### Data analysis

LPA is a statistical classification method for investigating hidden groups in observed patterns that examines the probability that respondents belong to homogeneous subpopulations [40]. To conduct LPA, we used Mplus version 7.4 [41] offering model fit indices to determine the best latent model with the optimal number of tutors’ effectiveness profiles. First, we examined the Akaike Information Criterion (AIC) and the Bayesian Information Criterion (BIC). Lower AIC and BIC values indicate that the *k* cluster model fits better than the *k-1* cluster model [42]. Second, a significant *p*-value of the Lo-Mendell-Rubin Likelihood Ratio Test (LMR) [43] indicates that the *k* cluster model improves the fit over the *k-1* cluster model. Third, the entropy was evaluated for cluster classification uncertainty. As entropy values approach one, they imply a more clear classification of clusters [44]. Last, the sizes of cluster membership in the *k* cluster model were considered. Each cluster comprising less than 5% of the entire sample could be detrimental to the interpretability of the *k* cluster model [45]. We used multiple one-way analyses of covariance (ANCOVA) to investigate whether the blended PBL tutors’ effectiveness profiles perceived by medical students were associated with learning engagement, controlling for performance self-efficacy as a covariate. Follow-up post hoc tests were conducted to investigate specific cluster differences using the Bonferroni method because the assumption of equal variances was met.

### Ethics approval

Before the study, we received Institutional Review Board (IRB) approval from Chonnam National University (IRB No. 21040198-200701-HR-072-02). Before conducting this study in December 2021, we provided all information about this study in a written form that was available to students in PBL classrooms at CNUMS. Again, we offered the precise information about this study and the informed consent form electronically via a downloadable link prior to an online survey. After obtaining students’ consent, they were allowed to complete an online survey questionnaire in which no sensitive personally identifiable information was asked. Students understood that their participation was voluntary and were allowed to withdraw from this study without any disadvantages at any time. Also, students were informed that their responses to the survey questionnaire would be kept confidential and analyzed anonymously.

## Results

### Descriptive statistics of variables

Table 2 displays the means, standard deviations, and correlation coefficients of all variables used in this study.

**Table 2:** Means, standard deviations, and correlation coefficients of variables (*n* = 238).

Variables^a^	1	2	3	4	5	6	7	8	9
1. Constructive learning	-								
2. Self-directed learning	.81**	-							
3. Contextual learning	.82**	.79**	-						
4. Collaborative learning	.69**	.72**	.73**	-					
5. Interpersonal behavior	.68**	.66**	.72**	.73**	-				
6. Behavioral engagement	.59**	.53**	.55**	.47**	.47**	-			
7. Emotional engagement	.51**	.44**	.48**	.46**	.49**	.63**	-		
8. Cognitive engagement	.53**	.50**	.53**	.49**	.50**	.71**	.72**	-	
9. Performance self-efficacy	.21**	.21**	.22**	.14*	.22**	.27**	.29**	.30**	-
**Mean**	4.17	4.16	4.14	4.04	3.88	4.15	3.59	3.81	5.30
**Standard deviation**	.66	.74	.72	.74	.86	.57	.72	.71	1.85

*Note*: *p < .05

**p < .01

^a^Possible range for tutor effectiveness and learning engagement: 1–5 and performance self-efficacy: 1–9.

### Optimal class number related to the effectiveness profiles of tutor performance

As illustrated in Table 3, the model fit statistics were examined to determine the optimal latent class model with the effectiveness profiles of tutor performance in the blended PBL. As AIC and BIC values are gradually reduced, an elbow point of the scree plot for AIC and BIC is apparent at the 3-latent class model, which assumed that the 3-latent class model could have a relatively better model fit over the 2-latent class model. An insignificant *p*-value of the LMR test for the 4-latent class model indicates that the 4-latent class model would not have a better fit than the 3-latent class model. The entropy value of the 3-latent class model showed the closest value compared to the other latent class models. The distributions of class sizes for the 3-latent class model are not problematic for the interpretability of this latent class model. Therefore, we concluded that the 3-latent class model has optimal latent class profiles with the observed data of tutor performance in the blended PBL.

**Table 3:** Model fit statistics for the latent class models of tutor effectiveness profiles (*n* = 238).

Model	No. of parameters	AIC	BIC	LMR *p* value	Entropy	Optimal model 3—class counts (%)
2	34	4959.10	5077.16	.028	.973	24 (10.1%) 136 (57.1%) 78 (32.8%)
3	46	4165.21	4324.93	.022	.990	
4	58	4050.80	4252.20	.330	.985	
5	70	3892.06	4135.12	.426	.974	

### Characteristics of tutor effectiveness profiles in the optimal latent class model

**Fig 1:**
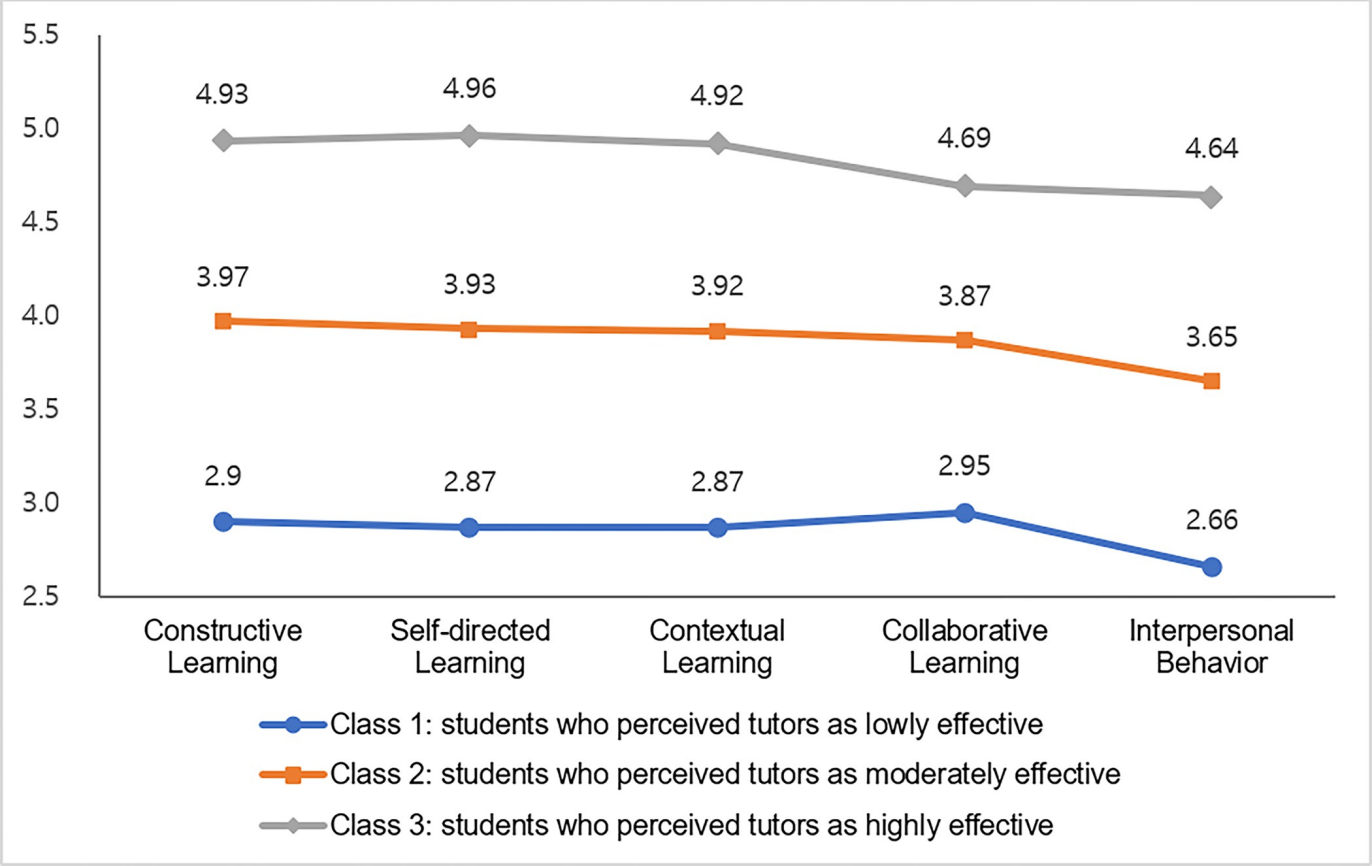
Tutor effectiveness profiles for the 3-latent class model.

The three tutor effectiveness profiles according to the variations of their performance perceived by medical students in the blended PBL were identified, as shown in Fig 1. Of the 238 students, 24 (10.1%) were assigned to the first class which revealed that tutor performance was regarded as less effective than any other classes across all learning factors. We labeled this class as students who perceived their tutors as lowly effective. The second class includes 136 (57.1%) students who assessed that the effectiveness of tutor performance was moderate at the medium levels in all learning factors. We labeled this class as students who perceived their tutors as moderately effective. The last 78 (32.8%) students indicated that tutor performance was the most effective in all learning factors and perceived that tutor performance in constructive learning, self-directed learning, and contextual learning was close to the highest level. We labeled this class as students who perceived their tutors as highly effective.

### Relationships between tutor effectiveness profiles and learning engagement

Multiple one-way ANCOVA were performed to determine whether the three types of learning engagement differ between the three latent profiles after controlling for students’ performance self-efficacy. As seen in Table 4, the results showed that these latent profiles have significant effects on learning engagement after controlling for the effect of performance self-efficacy (for behavioral engagement, *F*(2, 233) = 72.60, *p* < .001, partial η^2^ = .38; for emotional engagement, *F*(2, 233) = 33.64, *p* < .001, partial η^2^ = .22; for cognitive engagement, *F*(2, 233) = 37.87, *p* < .001, partial η^2^ = .24, respectively). The covariate, medical students’ performance self-efficacy, was significantly related to learning engagement (for behavioral engagement, *F*(1, 233) = 7.74, *p* = .006; for emotional engagement, *F*(1, 233) = 12.17, *p* = .001; for cognitive engagement, *F*(1, 233) = 12.83, *p* < .001, respectively). The post hoc tests using the Bonferroni method presented specific group comparisons.

**Table 4:** Results of one-way ANCOVA.

Outcome variables	Latent classes	M (SD)	Adjusted M	*F*	*P* value	Partial eta^2^	Post-hoc tests
Behavioral Engagement	Class 1	3.55 (.62)	3.58	72.60	< .001	.38	Class 3 > Class 2*** Class 3 > Class 1*** Class 2 > Class 1***
	Class 2	3.98 (.43)	3.99				
	Class 3	4.65 (.37)	4.62				
Emotional Engagement	Class 1	3.03 (.57)	3.09	33.64	< .001	.22	Class 3 > Class 2*** Class 3 > Class 1*** Class 2 > Class 1*
	Class 2	3.41 (.60)	3.42				
	Class 3	4.09 (.67)	4.05				
Cognitive Engagement	Class 1	3.15 (.66)	3.21	37.87	< .001	.24	Class 3 > Class 2*** Class 3 > Class 1*** Class 2 > Class 1**
	Class 2	3.64 (.54)	3.66				
	Class 3	4.30 (.69)	4.26				

*Note*: *p < .05

**p < .01

***p < .001; M: Mean, SD: Standard Deviation; Class 1: Students who perceived tutors as lowly effective, Class 2: Students who perceived tutors as moderately effective, Class 3: Students who perceived tutors as highly effective.

## Discussion

This study found that the effectiveness of tutor performance perceived by medical students in the blended PBL was classified into three different profile groups: lowly, moderately, and highly effective tutors. Overall, students became more engaged in the learning process if they felt that tutor performance was more effective. The effectiveness of tutor performance that students perceived during PBL activities was characterized based on the profiles of tutor effectiveness. For example, highly effective tutors that students recognized received the highest scores across all five learning factors. The scores of constructive, self-directed, and contextual learning were relatively higher than those of collaborative learning and interpersonal behaviors.

This finding provides supporting evidence for effective tutor performance, similar to findings from prior research. Tutor performance differed across different levels, which was associated with different levels of tutorial group productivity [46]. Effective tutors facilitated constructive learning through several approaches such as discussion, note-taking, or questioning that encourage students to actively participate in the learning process [28,34]. Furthermore, these tutors helped students become self-directed and internally goal-driven learners to engage in understandings the clinical learning process [47]. To foster contextual learning, they were assumed to provide a well-defined PBL task in which students could share various perspectives to improve their learning experiences [34,48].

Although tutors in the blended PBL process effectively supported constructive, self-directed, and contextual learning, students might struggle in the collaborative learning experience. According to a prior study [49], online PBL negatively affected collaborative learning due to the lack of physical presence and increased cognitive load compared to face-to-face PBL. Hence, mentoring was highly recommended to help passive participants overcome psychological difficulties and fears in the online PBL environment [49]. Moreover, Pires et al. [50] suggested that tutors need to support collaborative learning using elaboration strategies, which elicit integral concepts from different sources and prior experiences to acquire new knowledge. On the other hand, tutors should take students’ prior knowledge into account as designing a structured PBL curriculum because students’ performance in learning activities was crucially dependent on their prior learning experiences [51,52].

In addition, tutors need to show their commitment to students in the PBL settings to improve psychological safety so students are less likely to face conflicts [53]. That is, tutors should create a healthy and stable PBL environment for students so positive interpersonal interactions help facilitate group dynamics [54]. Likewise, students may feel comfortable participating in the blended PBL process if the sound social relationships with tutors and other participants in small groups are shaped. Furthermore, the Community of Inquiry (CoI) framework can be applied to enhance meaningful blended PBL with three individual disciplines: teaching presence, social presence, and cognitive presence [55]. Since the CoI framework places a strong emphasis on students’ interaction within a socio-cultural context, tutors consciously need to create a student-centered learning community where students reflect on the learning process and establish group cohesion [56]. Thus, tutors must build opportunities for social interactions and positive rapport with students and create a friendly learning climate [53].

Next, this study confirmed that the effectiveness of tutor performance was significantly associated with positive learning outcomes, which supports previous findings [57]. We found that students who perceived highly-effective tutor performance had higher levels of three types of learning engagement. Alimoglu et al. [57] accentuated that instructors’ characteristics, relationships with students, and communications are critical external factors that affect student engagement in PBL. The effectiveness of tutor performance is influential in encouraging students to engage behaviorally and cognitively during the PBL process. However, tutors need to improve students’ emotional engagement. When students have tutors who develop a good rapport and interpersonal relationships with students, they could become highly engaged in learning [58]. Students may experience more autonomous motivation related to learning [59]. Thus, tutors should support students to become intrinsically motivated to learn.

Medical institutions must provide training programs as part of an effective PBL curriculum [1,14]. Training programs incorporating tutor shadowing known as peer observation can help novice PBL tutors develop competencies [60]. Hands-on training support for novice PBL tutors who lack social and interpersonal skills can enable a successful PBL program [17]. This study highlighted the importance of tutors facilitating learning engagement in the blended PBL process. Students feel warmth and social congruence when tutors consistently interact with them during the PBL process [58]. Fostering students’ interest and enjoyment of learning is an important factor for a successful PBL experience [20]. Regarding the integration of technology in a PBL curriculum, the use of mobile devices (e.g., iPads with applications) in a hybrid PBL medical curriculum encourages student engagement but also alters the PBL process [14].

This study has some limitations. First, the findings from this study cannot be generalizable because this blended PBL curriculum was contextualized in a specific medical education setting at CNUMS and a limited number of medical students participated in this study. Future research should involve a large-scale longitudinal study to investigate the effectiveness of tutor performance at national levels in medical education. The second limitation is the first-year and second-year medical students in the sample may challenge the interpretation of our results. The two different groups of participants may have differences in several individual factors, such as experiences with PBL or blended learning, motivation to participate in PBL, attitudes toward and perspectives of tutors, and relationships with tutors. Participants’ backgrounds should be examined. Lastly, employing a cross-sectional design, this study relies on self-reported survey data that assessed medical students’ perceptions of tutor performance with no direct indication of causal relationships among variables. Focus-group interviews and observations may provide a deeper understanding of tutor effectiveness in blended PBL.

## Conclusions

This study provided empirical evidence of medical students’ perceptions of tutor performance and learning engagement was highly associated with the effectiveness of tutor performance in the blended PBL process. Highly engaged students in blended PBL appeared to recognize that tutors were highly effective in promoting constructive, self-directed, and contextual learning. They need to complement collaborative learning and foster conducive interpersonal behaviors in students. Conversely, better performing students might be less demanding towards tutor support. Further research utilizing longitudinal data collection methods is needed to measure changes over time. Meanwhile, this study highlights that the tutor is one of the key aspects of assessing the implementation of blended PBL, since student performance is positively associated with the perception of tutor effectiveness.
